# The green view dataset for the capital of Finland, Helsinki

**DOI:** 10.1016/j.dib.2020.105601

**Published:** 2020-04-22

**Authors:** Akseli Toikka, Elias Willberg, Ville Mäkinen, Tuuli Toivonen, Juha Oksanen

**Affiliations:** a Finnish Geospatial Research Institute FGI, National Land Survey of Finland; b Digital Geography Lab, Department of Geosciences and Geography, University of Helsinki; c Helsinki Institute of Sustainability Science, University of Helsinki

**Keywords:** Green View Index, Street View, Helsinki, Human aspect, City greenery

## Abstract

Recent studies have incorporated human perspective methods like making use of street view images and measuring green view in addition to more traditional ways of mapping city greenery [1]. Green view describes the relative amount of green vegetation visible at street level and is often measured with the green view index (GVI), which describes the percentage of green vegetation in a street view image or images of a certain location [2]. The green view dataset of Helsinki was created as part of the master's thesis of Akseli Toikka at the University of Helsinki [3].

We calculated the GVI values for a set of locations on the streets of Helsinki using Google Street View (GSV) 360° panorama images from summer months (May through September) between 2009 and 2017. From the available images, a total of 94 454 matched the selection criteria. These were downloaded using the Google application programming interface (API). We calculated the GVI values from the panoramas based on the spectral characteristics of green vegetation in RGB images. The result was a set of points along the street network with GVI values.

By combining the point data with the street network data of the area, we generated a dataset for GVI values along the street centre lines. Streets with GVI points within a threshold distance of 30 meters were given the average of the GVI values of the points. For the streets with no points in the vicinity (∼67%), the land cover data from the area was used to estimate the GVI, as suggested in the thesis [3]. The point and street-wise data are stored in georeferenced tables that can be utilized for further analyses with geographical information systems.

Specifications table

**Table:** 

Subject	Computers in Earth Sciences
Specific subject area	Mapping city greenery through street view imagery.
Type of data	Tables with georeferenced coordinates.
How data were acquired	Google Street View (GSV) 360° panoramas were acquired and analysed using open source programming tools with Python. Each panorama contains six images taken towards the following headings: 0°, 60°, 120°, 180°, 240°, and 300°.
Data format	Raw and analysed.
Parameters for data collection	Only GSV panoramas taken between 2009 and 2017 during May through September (leaf-on conditions) were used in the analyses. Panoramas were not acquired from motorways, walkways or bridleways of open street maps.
Description of data collection	Metadata of GSV panoramas of Helsinki city area was downloaded from Google API. Based on the metadata, only the panoramas taken during summer months were downloaded. Each panorama was downloaded and analysed in six horizontal images. The final GVI value assigned to the panorama was the mean value of these six images. The street-wise GVI values are either the mean of the GVI values of nearby GVI points or estimated from the land cover data from the study area.
Data source location	Helsinki, Finland
Data accessibility	With the article

## Value of the data

• Instead of the traditional overhead view, the GVI dataset presents the human-level aspect of the distribution of city greenery in Helsinki.
• The dataset can be beneficial for decision makers and urban planners.
• The dataset can be used in various studies, for example those investigating the effects of city greenery on human physical and mental health. It can also be beneficial when planning more pleasant cycling and pedestrian paths and neighbourhoods.
• When combined with other existing city greenery datasets, the green view dataset can help to build a more holistic understanding of the city greenery in Helsinki.

## 1 Data

**Fig. 1:**
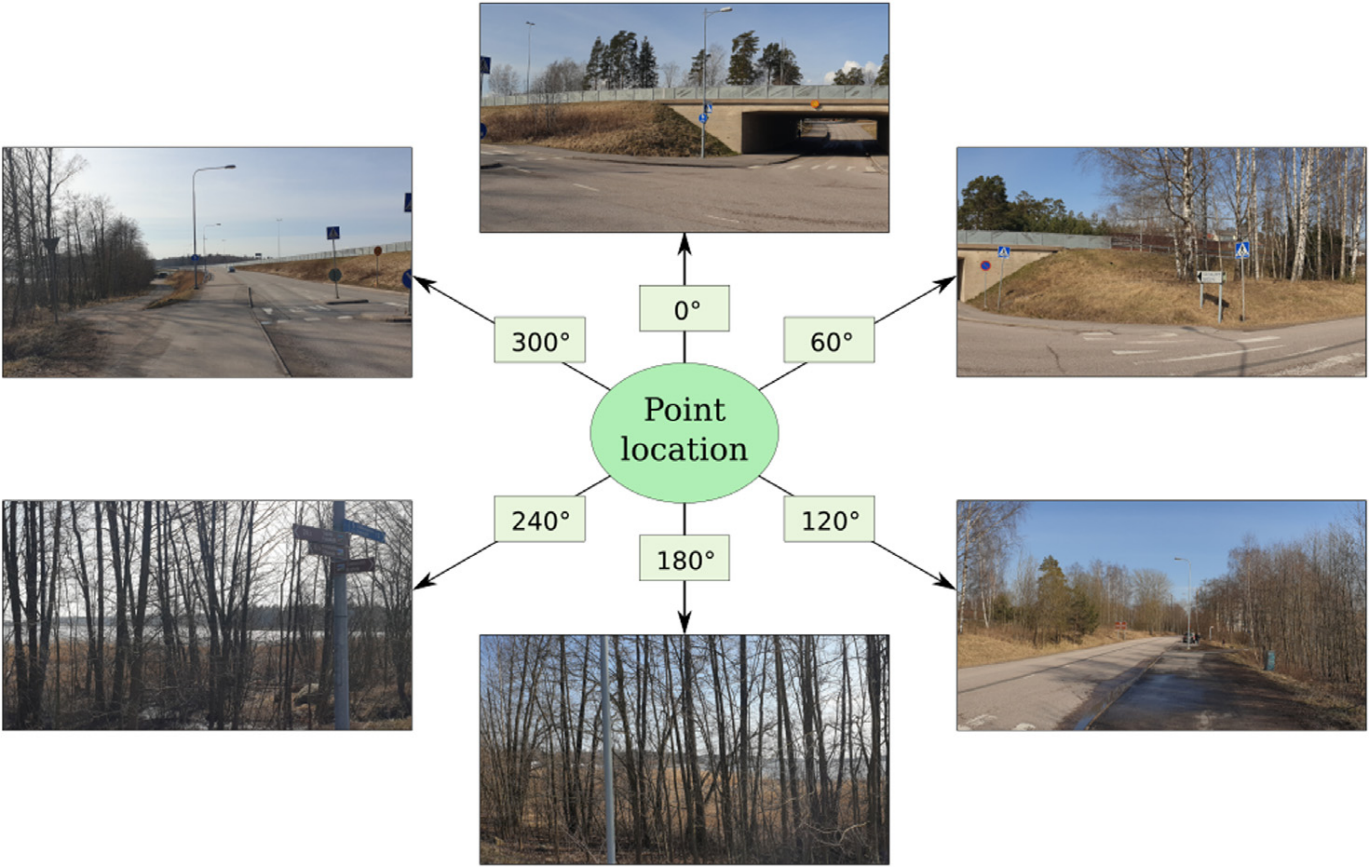
Each GSV panorama was downloaded as six horizontal images and named after the heading. Note: Images are self-taken and they simulate here the copyrighted GSV images.

**Fig. 2:**
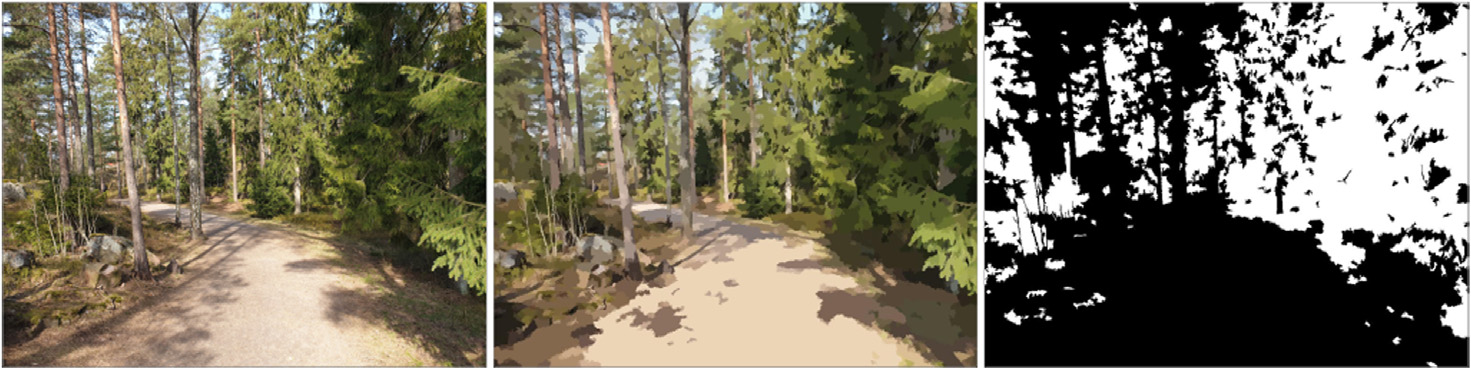
A. Left. The original image. Middle: The segmented image Right: The final binary image in which the white areas represent vegetation. The GVI of this image is 38%. Note: The image is self-taken and it simulates here the copyrighted GSV images.

**Fig. 3:**
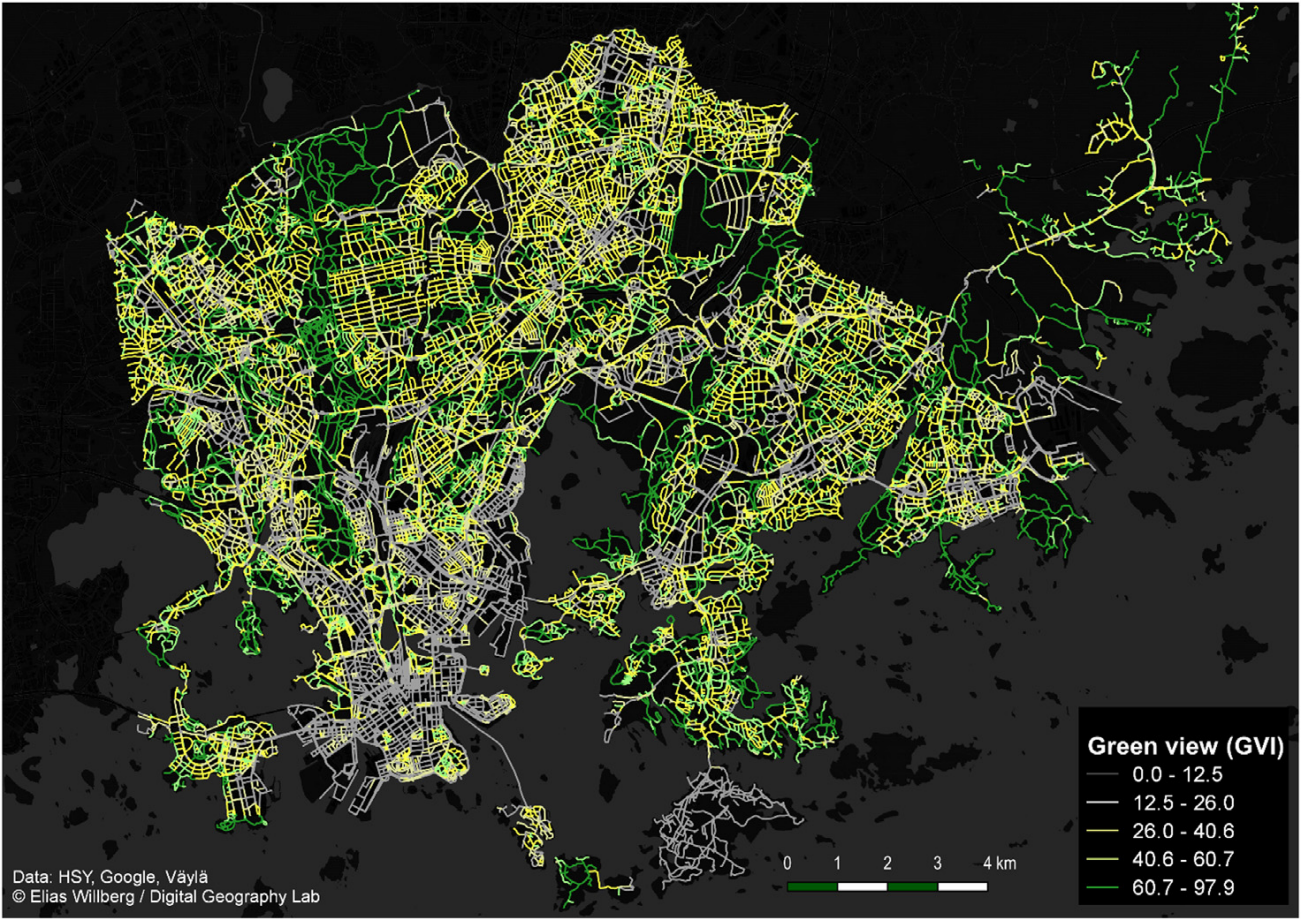
The variation of green view index values in the Helsinki street network.

The two datasets in this article describe the GVI of the city of Helsinki, Finland, determined using GSV images. The datasets are in GeoPackage format, which is an open standard format for transferring geospatial information. The dataset “greenery_points.gpkg” contains points, located on the street network of the area, for which the GVI was determined from the GSV images. All the attributes related to the points are listed in Table 1. The dataset “greenery_roads.gpkg” contains the line features of the street network dataset [9], enriched with GVI values calculated from the nearby points from the dataset “greenery_points.gpkg” and from the land cover data [12]. The attributes related to the street geometries are listed in Table 2. Fig. 1. shows the format to which the GSV images were downloaded. Fig. 2. illustrates the method that was used to determine the amount of vegetation in the images. Fig. 3. visualizes the GVI values of the road network in the dataset “greenery_roads.gpkg”.

**Table 1:** Descriptions of the point dataset column titles.

Column name	Column description
panoID	Unique ID of the panorama image by Google
panoDate	Year and month in which the panorama was taken
longitude	Panorama location longitude in WGS 84
latitude	Panorama location latitude in WGS 84
GviH_0	GVI of the image taken towards the heading 0°
GviH_60	GVI of the image taken towards the heading 60°
GviH_120	GVI of the image taken towards the heading 120°
GviH_180	GVI of the image taken towards the heading 180°
GivH_240	GVI of the image taken towards the heading 240°
GviH_300	GVI of the image taken towards the heading 300°
Gvi_Mean	The mean GVI of the 6 images (= panorama GVI)

**Table 2:** Descriptions of the street network dataset column titles.

Column name	Column description
FID	Unique ID of the street segment
TEKSTI	Street name in Finnish
TOIMINNALL	Functional class
TYYPPI	Type of street segment
LIIKENNEVI	Direction of traffic in relation to direction of digitization
Luokka	Road class (including walking and cycling streets)
Pyoravayla	Cycleway
Shape_Leng	Segment length
GSV_GVI	Street view-based green-view index
BufArea	Area of the 30m buffer around the segment in m^2^
LUArea	Area of the tree cover within the buffer in m^2^
LU_GVI	Land use-based green-view index
Comb_GVI	Combined street view- and land use-based green view index
GVI_source	Source of green-view index (GSV or land use data)

### 1.1 GVI point data from GSV images (greenery_points.gpkg)

The green view point dataset of Helsinki is stored in a GeoPackage where every row contains information on the amount of vegetation in one GSV panorama. The column descriptions are presented in Table 1. The dataset contains information on the 94 454 panoramas downloaded from Google API. The coordinate reference system of the file is WGS 84 (EPSG: 4326).

### 1.2 Street-wise GVI data (greenery_roads.gpkg)

The green view network dataset of Helsinki is stored in a GeoPackage where every row represents a single street segment and contains the GVI index. The column descriptions are presented in Table 2. The coordinate reference system of the file is WGS 84 (EPSG: 4326). Beside green-view related columns, the dataset includes columns that allow routing operations with the dataset (id, street name, road class, functional class, traffic direction, type of the street segment and binary cycleway column).

## 2 Experimental Design, Materials, and Methods

### 2.1 GVI point data

The data was acquired and analysed using Python programming language and libraries. The scripts used in the process can be found at the GitHub repositories Helsinki_GreenView and Helsinki_GreenNetwork.

The first step of the process was to determine the relevant locations for GSV panoramas. The streets from OpenStreetMap within the Helsinki region (excluding motorways, walkways and bridle paths) were used as the input street network data. The line network was transformed into points with a minimum distance of 20 m, using the script CreatePoints.py.

After generating the point data, the Google API was queried to find GSV images near the points. The querying was done with several URL requests by using the script MetadataCollector.py. If a panorama image existed within 50 m of the points, the metadata of that panorama was saved in a text file. The metadata contains panorama ID, month/year in which the image was taken, and the spatial coordinates.

For the analysis, only the images where the foliage is green were relevant. Therefore, only the panoramas that were taken between May and September were downloaded, using the script GSV_image_downloader.py. Every panorama was downloaded in six horizontal images (Fig. 1) with a set of URL requests with the parameters of: “pano” = panorama ID of the image, “size” = 400*400 pixels, “fov” (field of view) = 60° and “pitch” = 0°. The clockwise direction of the view (parameter “heading”) was either 0° (North), 60°, 120°, 180°, 240° or 300°. The images were saved and named after the panorama ID and heading.

To calculate the GVI values for the relevant locations, each downloaded image needed to be processed. The first step of image processing was to segment the image into homogeneous clusters. This was achieved with the Mean Shift segmentation algorithm [4] with the following parameters: spatial radius = 6 pixels, range radius = 7, and minimum size of the cluster = 40 pixels (Fig. 2B). After segmentation, pixel values were scaled to a range between 0 and 1 by dividing the pixel values by 255.

The number of pixels representing vegetation in the segmented image was calculated by using the “excess green” (ExG) vegetation index [5,6] and Otsu's threshold method [7]. Two binary images were calculated based on threshold values in light and shadow. Threshold values based on sample street greenery pixels were used to distinguish vegetation in different lighting conditions. An additional two binary images were calculated, one for light and one for shadow conditions, using the threshold values determined in the Treepedia project [8]. The four binary images were combined into one by multiplying the light and shadow images with each other and then summing up the resulting two images. This produced a binary image with vegetation pixels and non-vegetation pixels represented with values one and zero, respectively (Image 2C). Finally, the GVI for the *i*th image from location *p* is defined as

$$GVI_{p,i}=\frac{\text{Number}\;\text{of}\;\text{vegetation}\;\text{pixel}s_{p,i}}{\text{Number}\;\text{of}\;\text{pixel}s_{p,i}}$$

Using the GVI values of the downloaded images, the GVI for each panorama can be determined. The GVI for a panorama *p* is defined here as the average of the GVI values of the individual images from that location:

$$GVI_{p}=\frac{1}{6}\sum_{i=1}^{6}GVI_{p,i}$$

The results were compiled in a table by using the script GVI_to_shp.py, which saves all the result files as a georeferenced data file that is ready for further analyses.

### 2.2 Street-wise GVI data

To create the green view network for Helsinki, the GVI values from the georeferenced points were aggregated to the street network by using the Street_view_GVI_to_network.py and stored in the column GSV_GVI. The street network used in this work, the MetropAccess-CyclingNetwork [9], is based on national Digiroad data [10] and was further modified [11] to model walking and cycling in Helsinki.

For each street segment that had GVI points within 30 m, the GVI value for the segment was defined as the average of those GVI points. Since GSV images are from the car-accessible streets only, this operation produced a GVI index for 37 772 of the 56 904 segments.

The GVI for the segments lacking the street view-based index was calculated with the regional land use data [12] from Helsinki. The data contains polygons indicating the areas with trees in the city. In the thesis [3], it was shown that the street segment-wise GVI values were strongly correlated with the amount of tree cover in the nearby area. Therefore, for the street segments without GVI points in their vicinity, their GVI values were estimated as the fraction of the tree cover in their neighborhood:

$$LU\_GVI_{s}=\frac{\text{Total}\;\text{area}\;\text{of}\;\text{tree}\;\text{cover}\;\text{polygons}\;\text{within}\;\text{the}\;buffer_{s}}{\text{Total}\;\text{area}\;\text{of}\;\text{the}\;buffer_{s}}$$

where *buffer_s_* is the street segment *s* buffered with a 30 m area. The values are scaled to a range between 0–100% by the operation, consistent with the street view-based GVI values. These complementary LU_GVI values were calculated using the script Land_use_GVI_to_network.py and stored in the column LU_GVI. Finally, a combined GVI column (Comb_GVI) was created, where the street view-based GVI was applied (if available) for the segment, otherwise the value of the land-use based GVI is used. The resulting green view street network was saved to a file. Fig. 3 shows the GVI variation in the streets of Helsinki.

## Appendix Supplementary materials

Image, application 1

Image, application 2

Image, application 3
